# The Beneficial Effect of *Lycium barbarum* Polysaccharides on Insulin Resistance and Hepatic Glucose Production in Diabetes

**DOI:** 10.1155/ancp/3176140

**Published:** 2025-09-15

**Authors:** Fengqi Wan, Jiong Dang, Shan Huang, Jiao Cai, Yu Lu, Jiaxin Wu, Jing Wang, Liang Ma

**Affiliations:** ^1^The Second Hospital and Clinical Medical School, Lanzhou University, Lanzhou, China; ^2^School of Pharmacy, Lanzhou University, Lanzhou, China; ^3^Institute of Modern Physics, Chinese Academy of Sciences, Lanzhou, China

## Abstract

*Lycium barbarum* polysaccharides (LBP) displays some antidiabetic effects, but the mechanism is partial disclosure of its preventive activities related to insulin signaling and hepatic glucose metabolism. This study investigates the beneficial effect of LBP on insulin resistance and hepatic glucose production (HGP) in 30 mM glucose induced insulin resistant HepG2 cells (IR-HepG2) and high fat diet/streptozotocin induced diabetic mice. Additionally, the subacute toxicity of 14-day-administration of LBP was assessed in C57BL/6 mice. The results showed that LBP effectively reverted the inhibition of protein kinase B (AKT) and glycogen synthase 3 (GSK3) phosphorylation and countered the elevation of reactive oxygen species (ROS) in IR-HepG2 triggered by 30 mM glucose. Furthermore, LBP prevented the decline of glucose transporter isoform 2 (GLUT2) level in the diabetic mice liver and restored reduced glucose consumption and uptake in IR-HepG2. LBP also prevented the decrease in glycogen synthase (GYS2) mRNA expression and the reduction of liver glycogen content in diabetes mellitus (DM) mice. Moreover, LBP downregulated the expressions of glucose-6-phosphatase (G6Pase) and phosphoenolpyruvate carboxykinase (PEPCK) to inhibit gluconeogenesis, while upregulated the expressions of glucokinase (GK), phosphofructokinase 1 (PFK1) and pyruvate kinase (PK) to activate glycolysis via the AMP-activated protein kinase (AMPK) signaling pathway in DM mice. No observed toxicity was found in both HepG2 cells and C57BL/6 mice under the tested conditions and doses. These findings suggest that LBP improves insulin sensitivity in high glucose induced IR-HepG2 and reduces HGP by regulating glucose uptake, glycogen synthesis and gluconeogenic/glycolytic flux. It may serve as a potential therapeutic approach for diabetes treatment.

## 1. Introduction

Diabetes mellitus (DM) is a chronic systemic metabolic disease and one of the most challenging healthcare epidemics in the world [1, 2]. Since that the biological injection of insulin or chemical oral drugs each have their limitations and drawbacks for treating DM, novel therapeutic approaches and functional food are therefore warranted [3–5]. Natural source polysaccharides have shown beneficial effects in diabetes treatment [6, 7], among them, *Lycium barbarum* polysaccharide (LBP), as the most abundant component of *LB*L., has been identified to influence the blood glucose homeostasis, lipid metabolism, and the altered oxidative and inflammatory status associated with diabetic complications [8–10].

The most underlying abnormality of type 2 DM is insulin resistance [11]. The protein kinase B (AKT) signaling is central to hepatocellular insulin action. Insulin binding to its receptor triggers tyrosine phosphorylation of insulin receptor substrates (IRS), activating the phosphoinositide-3-kinase(PI3K)/AKT pathway. AKT phosphorylation and subsequent inactivation of glycogen synthase 3 (GSK3) promote insulin-stimulated glycogen synthesis [12]. Previous studies have showed that LBP acts as a novel antioxidant against insulin resistance in palmitate-stimulated HepG2 via PI3K/AKT/nuclear factor E2-related factor 2 (Nrf2) pathway [13]. LBP also increases glucose consumption, possibly due to enhanced glucokinase (GK) and pyruvate kinase (PK) activities [14]. These properties suggest that LBP may offer protection against insulin resistance and regulate glucose metabolism in the liver. However, the comprehensive effect of LBP on insulin signaling and hepatic glucose production (HGP) remains unclear.

Here, we show the protective effect of LBP against insulin signaling restraint induced by a high glucose challenge in HepG2 cells and investigate the regulation of LBP on HGP in HFD/STZ-induced DM mice. Key proteins in the insulin signaling pathway, as well as the networks of glucose uptake, glycogen synthesis, gluconeogenesis and glycolysis, were evaluated. Our findings suggest that LBP could improve insulin sensitivity and reduce the HGP, offering an alternative therapy for DM treatment.

## 2. Materials and Methods

### 2.1. Materials

LBP was extracted and purified from the fruit of *LB*L. using the method of water extraction and ethanol precipitation in our laboratory. It is composed of arabinose, galactose, glucose, galacturonic acid, mannose, and rhamnose at a molar ratio of 12.25:8.66:7.66:2.86:1.70:1.00 (Figure S1) [15]. Liver tissues used in this study are samples from mice of control, diabetic, MET (metformin, 400 mg/kg body weight), and LBP (80 mg/kg body weight) groups from our previous research [16]. Animal studies were approved by the Animal Research Committee of Lanzhou University Second Hospital (Number D2019-069). Mice were fed with high-fat diet for 3 months and administrated intraperitoneally with streptozotocin to induce hyperglycemia (blood glucose >11.1 mM). Diabetic mice were treated by gavage daily with MET (400 mg/kg body weight), LBP (40, 80, and 160 mg/kg body weight), or saline for 8 weeks. The medium concentration of LBP (80 mg/kg body weight) was found to be the best effective dosage for decreasing blood glucose and alleviating insulin resistance in diabetic mice. Liver samples from this group were used to evaluate its beneficial effect on hepatic glucose production. The procedure is summarized in the schedule 1 of Figure S2.

### 2.2. Cell Culture and Induction of Insulin Resistance HepG2 Cell Model

HepG2 cells were obtained from national collection of authenticated cell cultures of China. Cells were cultured in low glucose Dulbecco's modified Eagle medium (DMEM containing 5.55 mM glucose; Gibco, China) with 10% fetal bovine serum (FBS; PAN, Germany). High glucose concentration in the medium is a common method to induced insulin resistant HepG2 (IR-HepG2) model [17]. In our study, 30 mM glucose was used to induced IR-HepG2 cells. Cells were incubated in serum-free DMEM containing either 5.55 or 30 mM D-glucose for 24 h. Before harvesting, cells were stimulated with 100 nM insulin (Solarbio, China) for 10 min [18–20]. Total cell extract was analyzed for the phosphorylation of AKT and GSK3α/β by immunoblots.

### 2.3. Cell Viability

About 5000 cells were seeded on 96-well plate. Normal and IR cells were treated with different concentrations of LBP (10, 25, 50, and 100 μg/mL) for 24 h. CCK8 solution (biosharp, China) was added, and after a 2-h incubation at 37°C, absorbance was measured at 450 nm using a microplate reader (VARIOSKAN FLASH, Thermo Scientific).

### 2.4. Reactive Oxygen Species (ROS) Detection

The level of intracellular ROS was monitored by DCF fluorescence method [21, 22]. 5000 cells were seeded in the 96-well plate and incubated with different concentrations of LBP (10, 25, 50, and 100 μg/mL) in 30 mM glucose condition for 12, 24, and 48 h, using 2 mM MET (Solarbio, China) as the positive treatment. Then discarded the medium and washed the cells twice with PBS. Cells were incubated with 10 μM 2′, 7′-dichlorodihydrofluorescein (DCFH-DA; Beyotime, China) in FBS free DMEM medium at 37°C for 25 min. After washing the cells three times with FBS free DMEM medium, 100 μL PBS with 2% FBS was added, and the fluorescence intensity of DCF was measured using a microplate reader at an excitation wavelength of 485 nm and an emission wavelength of 525 nm. Additionally, 2 × 10^5^ cells were seeded on 6 well culture plate, and the treatment conditions were the same as above. After incubation for 48 h, cells were harvested and suspended in 500 μL PBS with 2% FBS for flow cytometry analysis (CytoFLEX, Beckman).

### 2.5. Glucose Consumption and Glucose Uptake Assays

Cells were seeded into a 12-well plate at the density of 2.5 × 10^5^ per well and incubated with LBP (at concentrations of 10, 25, 50, and 100 μg/mL) in the presence of 30 mM glucose for 24 h. The medium was then discarded and serum free DMEM was added to each well for an additional 6 h. Glucose in the medium of each well was measured using the Glucose Assay Kit (Invitrogen, USA) with the glucose oxidase method [23]. Glucose consumption was calculated by the glucose concentrations in supernatant of blank wells (no cell) minus those in plated wells and normalized to the cellular protein concentration measured with the BCA Protein Assay Kit [24].

Glucose uptake was assessed using the fluorescent glucose analog: 2-(N-(7-nitrobenz-2-oxa-1,3-diazol-4-yl) amino)-2-deoxy-D-glucose (2-NBDG; Beyotime, China) [25]. HepG2 cells were seeded in 6 wells plate (5 × 10^5^ per well). After treatment, cells were harvested in glucose-free DMEM medium (Gibco, USA) to stain with 150 μM 2-NBDG at 37°C for 30 min. Cells were then washed twice with PBS and the fluorescence intensity was immediately detected by flow cytometry.

### 2.6. Subacute Toxicity Test

Although no adverse effects of LBP have been demonstrated until now, there is little scientific evidence about its toxicological effects. In this study, we evaluated the subacute toxicity of a 14-day continuous gavage of LBP at a dose of 2 g/kg body weight in C57BL/6 mice. Mice were randomly assigned to two groups: control and LBP (*n* = 10 mice/group). LBP was administered orally once daily, while the control group received an equal volume of saline. The Irwin test was employed to evaluate potential effects on the central nervous system, including assessments of excitation, motor activity, sedation, pain, autonomic effects, and respiration [26].

After 14 days of treatment, animals were deeply anesthetized, and blood was collected for hematological parameters (BC-6000Plus, mindray, China) and serum biochemical analyses (AU5800, Beckman Coulter). The liver, heart, kidneys, lungs, and spleen were removed, cleaned and weighted to determine relative organ weights (organ weight × 100/body weight) [27, 28]. Tissue samples from these organs were fixed in 10% neutral buffered formalin and embedded in paraffin for histopathological examination. Sections were dyed with hematoxylin and eosin (HE; Solarbio, China) according to the procedure of our previous study [16]. The procedure is summarized in the schedule 2 of Figure S2.

### 2.7. Quantitative Real-Time Polymerase Chain Reaction (qRT-PCR)

Total RNA was extracted from the liver using Trizol reagent (Takara, Japan). RNA concentration was assessed using a spectrophotometers (Nanodrop 2000, Thermo Fisher). RNA was reverse-transcribed to cDNA (Catalog Number: RR047A, Takara, Japan). SYBR Green dye (Catalog Number: RR091A, Takara, Japan) and primers were used for quantitative RT-PCR amplification and the product detection was performed on a LightCycler 96 instrument (Roche). mRNA levels of all genes were normalized to the internal reference gene *β*-actin according to the 2^−ΔΔCT^ method. Primers sequences used for qRT-PCR are listed in Table S1.

### 2.8. Western Blot

Cells and liver tissues were lysed with radioimmunoprecipitation buffer (RIPA, containing protease and phosphatase inhibitors) and centrifuged at 12,000 × *g* for 15 min. Protein lysates were separated by SDS/PAGE, blotted onto a PVDF membrane, and probed overnight at 4°C with primary antibodies (1:1000 dilutions for target proteins and 1:5000 for GAPDH), anti-GAPDH (Catalog Number: 10494-1-AP, proteintech, USA), anti-p-AKT and anti-p-GSK3α/β (Catalog Number: 4060 and 8566, CST, USA); anti-AKT and anti-p-AMP-activated protein kinase (AMPK) *α*1/2 (Catalog Number: ab179463 and ab133448, abcam, USA), anti-GSK3α/β, anti-glucose transporter isoform 2 (GLUT2), anti-AMPKα1/2, and anti-phosphoenolpyruvate carboxykinase (PEPCK) (catalog number: sc-7291, sc-518022, sc-74461, and sc-271029, SANTA CRUZ, USA), anti-glucose-6-phosphatase (G6Pase) (Catalog Number: PA45-42541, Invitrogen, USA). Membranes were washed and re-probed with a secondary antibody of goat anti-mouse or anti-rabbit IgG (1:5000; proteintech, USA) and then visualized with enhanced chemiluminescence.

### 2.9. Glycogen, GK, phosphofructokinase 1 (PFK1), and PK Quantification in the Liver

Concentrations of glycogen and PFK1 (Catalog Number: A043-1-1 and H244-1-2, Nanjing Jiancheng, China), GK, and PK (Catalog Number: JL31594 and JL11938, J&l Biological Co., China) in the liver were measured with commercial assay kits according to the manual books.

### 2.10. Statistical Analysis

Statistical analyses were performed using Graphpad Prism 7 software. All data were expressed as the mean ± standard deviation (SD). Normality was assessed using the Shapiro–Wilk test. In terms of data with normality, two-tailed Student's *t* test for two groups and one-way analysis of variance (one-way ANOVA) plus post hoc Dunnett's test for comparisons of multiple groups were employed. For non-normally distributed data, Mann–Whitney test was performed. Differences were considered significant at *p* < 0.05.

## 3. Results

### 3.1. 30 mM Glucose Induced Insulin Resistance HepG2 Cells

**Figure 1:**
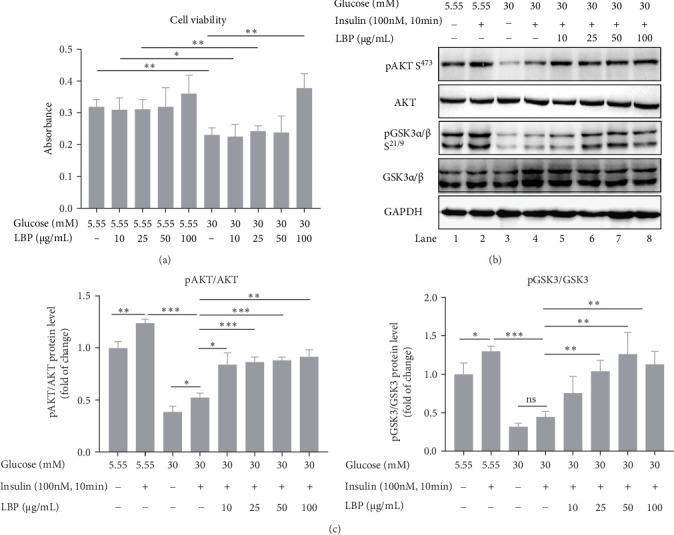
Effect of LBP on the viability and the activation of phosphorylated AKT/GSK3 in insulin resistant HepG2. (a) Effect of LBP on the viability of HepG2 cells under 5.55 or 30 mM glucose DMEM (*n* = 4 independent experiments), (b) representative western blot images of phosphorylated AKT/GSK3 and total AKT/GSK3, and (c) quantification of phosphorylated AKT/AKT and phosphorylated GSK3/GSK3 (*n* = 3 independent experiments). Values are means ± SD, *⁣*^*∗*^*p* < 0.05, *⁣*^*∗∗*^*p* < 0.01, and *⁣*^*∗∗∗*^*p<*0.001.

Treatment of HepG2 with LBP did not exhibit cytotoxicity. In fact, a concentration of 100 μg/mL LBP significantly alleviated the inhibition of HepG2 cell proliferation under 30 mM glucose conditions (*p* < 0.05, Figure 1a). Western blot analysis (Figure 1b–d) revealed that insulin (100 nM) markedly stimulated increases in AKT/GSK3 phosphorylation in HepG2 cells compared to the basal level (lane 1 and 2, *p* < 0.05). However, phosphorylation of AKT/GSK3 was significantly blocked under high-glucose conditions, indicating an insulin-resistant state (lane 4 and 2, *p* < 0.001). Notably, all three concentrations of LBP (25, 50, and 100 μg/mL) significantly enhanced AKT/GSK3 phosphorylations under high-glucose conditions when compared to the insulin-resistant group (lane 6, 7, and 8 vs., lane 4).

### 3.2. Effect of LBP on Intracellular ROS Content

**Figure 2:**
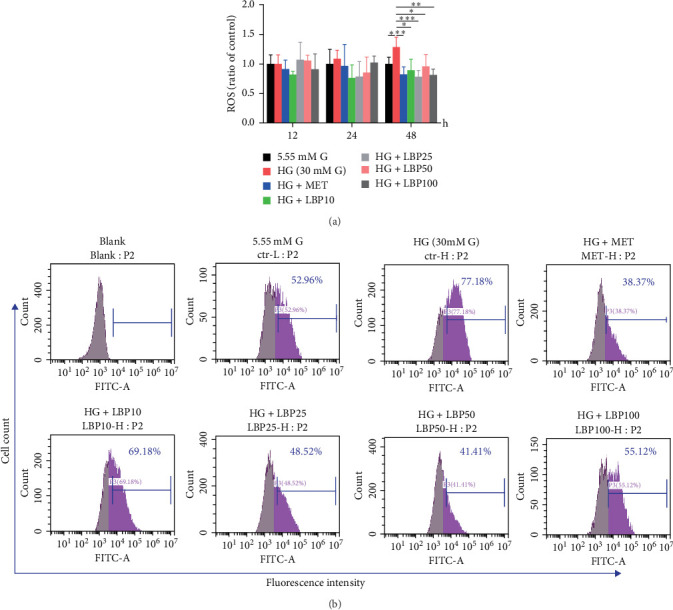
The scavenging effect of LBP on intracellular ROS in IR-HepG2 cells. (a) The time-dependent effect of LBP on the content of ROS was detected by fluorescence microplate, values are means ± SD, *n* = 3 independent experiments, *⁣*^*∗*^*p<*0.05, *⁣*^*∗∗*^*p<*0.01, and *⁣*^*∗∗∗*^*p<*0.001. (b) A representative experiment of intracellular ROS at 48 h was detected by flow cytometry. LBP10, LBP25, LBP50, and LBP100 means 10, 25, 50, 100 μg/mL LBP, respectively. MET means 2 mM metformin.

Insulin resistance and hyperglycemia are associated with oxidative stress, as evidenced by the increase in ROS levels [22, 29]. Under the condition of 30 mM glucose, the production of intracellular ROS increased with the prolongation of the culture time. At 48 h, ROS levels in IR-HepG2 were significantly increased by 28.68% compared to the control group, indicating that high glucose caused oxidative stress in HepG2 cells. When compared to the 30 mM glucose medium group, all four concentrations of LBP (10, 25, 50, and 100 μg/mL) significantly reduced the elevated intracellular ROS levels by 30.63%, 39.12%, 25.35%, and 36.68%, respectively (Figure 2a, *p*  < 0.05). This effect was confirmed by flow cytometry analysis, the proportion of ROS positive cells were reduced by 8%, 28.66%, 35.77%, and 22.06% in these four concentrations of LBP (Figure 2b).

### 3.3. Subacute Toxicity

**Figure 3:**
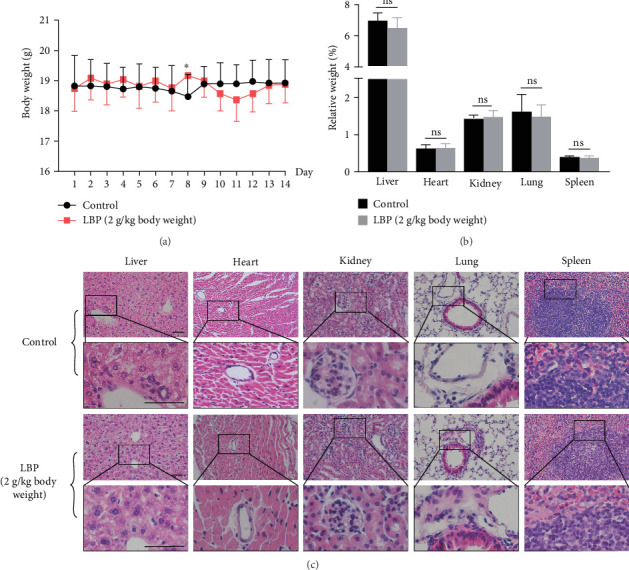
Body weight monitoring, relative weight and HE staining of vital organs in the subacute oral toxicity experiment. (a) Body weight monitoring during 14 days treatment, (b) relative organ weight of liver, heart, kidney, lung, and spleen at the end of 14 days treatment, and (c) representative HE staining sections of these organs (bar = 50 μm). Figures in the second row of each group is the enlarged sections from the first row. Values are means ± SD (*n* = 10 mice per group), *⁣*^*∗*^*p<*0.05, and ns means no significance between control and LBP groups.

LBP did not show any signs and features of toxicity or mortality at oral doses up to 2 g/kg body weight in C57BL/6 mice. Food and water consumption were monitored (Figure S3), and no alterations of body weight were observed in mice that received LBP or saline (Figure 3a). Behavioral (Table S2) and hematological (Table S3) parameters showed no significant differences between LBP-treated and control mice. No differences were noted between the two groups in biochemical parameters, such as serum glucose, total cholesterol, total protein, albumin, globulin, urea, creatinine, total bilirubin, and ions (K, Na, Ca, and Mg). However, triglyceride, ALT, and AST activities were significantly reduced by 32.56%, 23.53%, and 25.28%, respectively, with LBP treatment (Table S4). After 14 days of treatment, vital organs showed no alterations in relative weight or significant histological changes (Figure 3b, c).

### 3.4. LBP Promoted Hepatic Glucose Transport, Consumption, and Uptake

**Figure 4:**
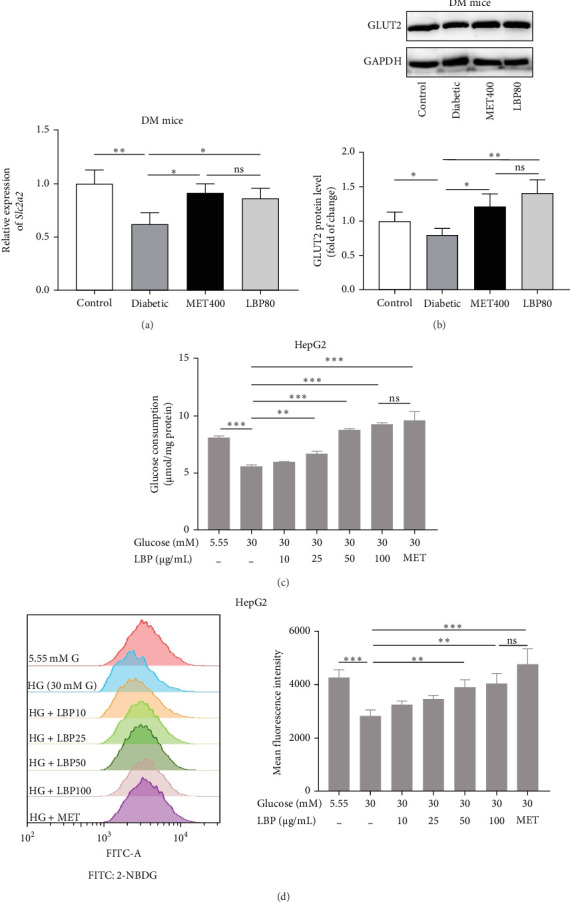
Effects of LBP on hepatic glucose transport, glucose consumption and glucose uptake in DM mice or IR-HepG2. (a) mRNA level of Slc2a2 in DM mice liver, (b) representative western blot and its quantification of GLUT2 protein level in DM mice liver, (c) dose–response of LBP on the glucose consumption of IR-HepG2, and (d) dose–response of LBP on the glucose uptake of IR-HepG2 measured by 2-NBDG. MET400 (diabetic mice treated with 400 mg/kg body weight metformin), LBP80 (diabetic mice treated with 80 mg/kg body weight LBP), and MET mean 2 mM metformin in HepG2 assays; Values are means ± SD, *n* = 3 independent experiments. *⁣*^*∗*^*p<*0.05, *⁣*^*∗∗*^*p<*0.01, *⁣*^*∗∗∗*^*p<*0.001, and ns means no significance.

*In vivo*, compared to the control group, the gene and protein expression levels of GLUT2 in DM mice were obviously downregulated by 37.92% and 20.17%, respectively. The administration of LBP and MET significantly reversed the downregulation of *Scl2a2* and GLUT2 expression compared to the diabetic group (1.39- and 1.77-fold for LBP treatment, 1.47- and 1.52-fold for MET treatment; *p<*0.05; Figure 4a, b). In vitro, 30 mM glucose remarkably inhibited glucose consumption and uptake in HepG2 by 31.06% and 33.84%, respectively. All three concentrations of LBP (25, 50, 100 μg/mL) significantly promoted glucose consumption in HepG2 in a dose-dependent manner, increasing consumption by 20.22%, 57.12%, and 65.95%, respectively compared to the 30 mM glucose condition (Figure 4c, *p<*0.05). Similar effects were observed in glucose uptake assay, where the two higher concentrations of LBP (50 and 100 μg/mL) enhanced the uptake of 2-NBDG in IR-HepG2, contributing to increases of 38.65% and 43.41%, respectively compared to the insulin resistant group (Figure 4d, *p<*0.05).

### 3.5. LBP Promoted Liver Glycogen Synthesis in DM Mice

**Figure 5:**
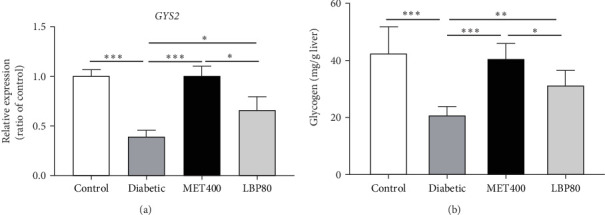
Effect of LBP on the liver glycogen synthesis of DM mice. (a) mRNA expression of *GYS2* in the liver (*n* = 3 independent experiments) and (b) the glycogen content in the liver. Values are means ± SD (*n* = 5–7 mice per group), *⁣*^*∗*^*p<*0.05, *⁣*^*∗∗*^*p<*0.01, and *⁣*^*∗∗∗*^*p<*0.001.

To further elucidate the hypoglycemic effects of LBP mediated by increased glycogen synthesis, the glycogen synthase (GYS2) mRNA level and liver glycogen content were measured for all groups after 8 weeks of gavage. The results indicated that, compared to the control group, the mRNA level of GYS2 and liver glycogen content in diabetic mice were significantly reduced by 61.35% and 51.09%, respectively (*p* < 0.001). LBP and MET obviously upregulated the expression of GYS2 by 68.43% and 160.54%, respectively, while the liver glycogen contents were 1.50- and 1.95-fold of changes, respectively, compared to the diabetic mice. These findings suggest that both LBP and MET possess the capability to promote glycogen synthesis in DM mice (Figure 5).

### 3.6. Effect of LBP on Hepatic Gluconeogenesis/Glycolysis Flux via Stimulating AMPK Phosphorylation in DM Mice

**Figure 6:**
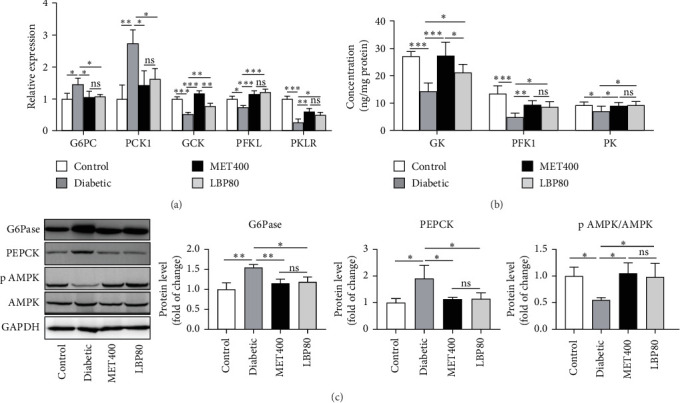
Effect of LBP on hepatic gluconeogenesis/glycolytic flux via stimulating AMPK phosphorylation in DM mice. (a) mRNA levels of G6PC, PCK1, GCK, PFKL, and PKLR (*n* = 3 independent experiments), (b) the contents of GK, PFK1, and PK tested by ELISA kits in the liver (*n* = 5–7 mice per group), and (c) representative western blot and its quantifications of G6Pase, PEPCK, and phosphorylated AMPK at Thr172 in the liver (*n* = 3 independent experiments). Values are means ± SD, *⁣*^*∗*^*p<*0.05, *⁣*^*∗∗*^*p<*0.01, *⁣*^*∗∗∗*^*p<*0.001, and ns means no significance.

In Figure 6, the results demonstrated that the mRNA levels of G6PC and PCK1 were significantly up-regulated, while *GCK*, *PFKL*, and *PKLR* were down-regulated in diabetic mice, which were 145.69%, 273.78%, 52.36%, 74.31%, and 26.84% of those indicators in the control group, respectively (*p<*0.05). The protein levels of G6Pase, PEPCK, GK, PFK1, and PK in diabetic mice were 154.70%, 191.00%, 52.89%, 36.40% and 75.17% of those indicators in the control group, respectively. Compared to the diabetic group, LBP intervention significantly reduced G6PC and PCK1 by 25.68% and 40.68% (*p* < 0.05), respectively, while increasing GCK, PFKL, and PKLR by 48.99%, 62.89% and 86.96%, respectively (*p* < 0.05). It also suppressed the protein level of G6Pase and PEPCK by 23.34% and 39.95%, respectively, while stimulating the expressions of GK, PFK1, and PK by 47.84%, 75.05%, and 32.94%, respectively, compared to the diabetic group. To elucidate the underlying mechanism of LBP, the phosphorylation of AMPK was examined. AMPK phosphorylation was inhibited in the diabetic group but was activated by LBP. All the above results indicated that LBP promoted the direction of gluconeogenesis/glycolysis flux toward glycolysis via activating the AMPK signaling pathway.

## 4. Discussion

Natural products have been proposed to play beneficial effects on health and have drawn attention because of their safety [30–32]. However, the composition of natural product extracts is different from the original herbs, necessitating a reevaluation of their toxicity [33]. In our subacute toxicity test, a dose of 2 g/kg body weight was administered, which is over 20 times higher than the effective concentration (80 mg/kg body weight). After 14-day-gavage, most of the tested hematological and biochemical parameters showed no significant differences between the LBP-treated group and the control group. Serum triglyceride was obviously reduced compared to the control group, which is consistent with the reported hypolipidemic effects of LBP [34–36]. For liver function test, the reductions of ALT and AST activities in LBP treatments were 23.53% and 25.28%, respectively, compared to the control group. Usually, elevations in ALT and AST disproportion to elevations in alkaline phosphatase and bilirubin denote hepatocellular disease [37]. However, reductions in ALT and AST within a certain range (< 50%) are generally considered to be “nonadverse” effects [38]. The subacute toxicity data in vivo along with the results from the cell viability assay in vitro, collectively suggest that LBP is reliably safe for pharmacological studies.

**Figure 7:**
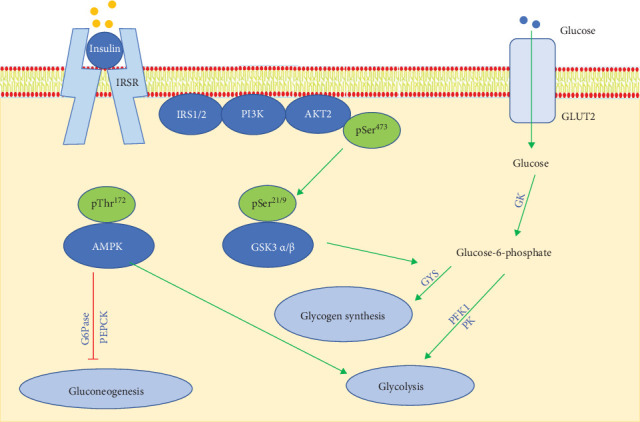
A schematic representation for the effect of LBP on hepatic insulin signaling and glucose production. LBP promotes GLUT2 to transport glucose from circulating blood to hepatocytes. As the glucose level rises, GK is activated to catalyze glucose to 6-phosphate glucose for glycogen synthesis and glycolysis. LBP induces the phosphorylation of AMPK at Thr 172, which can inhibit gluconeogenesis but enhance glycolysis by modulating G6Pase, PEPCK, GK, PFK1, and PK. In hepatic insulin signaling, LBP stimulates the phosphorylation of AKT at Ser 473. The activation of AKT leads to the inactivation of its substrates GSK3 by phosphorylating Ser 21 in the *α*-subunit and Ser 9 in the *β*-subunit. The inactivation of GSK3 increases the dephosphorylation of glycogen synthase, thereby promoting glycogen synthesis. Green arrows represent upregulating effect of LBP, while red arrows refer to the suppression effect of LBP.

The liver controls blood glucose by regulating glucose production and consumption through a balance of fluxes from glycogen synthesis, glycogenolysis, gluconeogenesis, glycolysis and other pathways [39, 40]. Glucose entrance into hepatocytes primarily via GLUT2 transporters and is then phosphorylated by GK to form glucose-6-phosphate (G6P). G6P can either be stored as glycogen or metabolized through glycolysis [41]. Out of 10 reactions in glycolysis, three are irreversible and involve specific enzymes that catalyze reactions either toward gluconeogenesis or glycolysis. Several transcription factors and coactivators have been identified to control the expression of these enzymes [42]. Among these regulators, AMPK is a critical regulator. Its activation improves hepatic insulin sensitivity and glucose homeostasis [24, 43]. LBP promotes glucose absorption for storage as glycogen or entry into the glycolysis process. It also downregulates the expression of G6Pase and PEPCK to inhibit gluconeogenesis, while upregulating the expression of GK, PFK1, and PK to enhance the glycolysis process through AMPK activation. Thus, our data demonstrate that the comprehensive effect of LBP on hepatic glucose metabolism is a reduction in HGP (Figure 7).

In our previous study, LBP showed weaker hypoglycemic effects than MET [16], possibly due to its weaker abilities to promote glycogen synthesis and upregulate GK. We have separated and purified five fractions from crude LBP [44]. Future research will identify which fraction reduces HGP, with the expectation of enhancing its hypoglycemic activity. In clinical practice, the combination of synthetic medicines and natural compounds is recognized as a strategy to improve efficacy and reduce side effects [30]. For example, combination therapies of MET with resveratrol or pholoretin have demonstrated synergistic effects against hyperglycemia and insulin resistance in various experimental models [45, 46]. Thus, further studies on combining LBP with MET are recommended to explore potential synergistic interactions.

In conclusion, the present study provides evidence for the antidiabetic effects of LBP and elucidates the related mechanisms. LBP showed no observed toxicity in both C57BL/6 mice and HepG2 cells under the tested conditions and doses. It exerts insulin-sensitizing effects by stimulating the phosphorylation of AKT (Ser 473) and GSK3 α/β (Ser 21/9), and by scavenging elevated intracellular ROS in IR-HepG2. Additionally, LBP promotes glucose transportation, consumption, uptake, glycogen synthesis, and glycolysis, while inhibiting gluconeogenesis. Collectively, these actions result in a reduction in hepatic glucose production.

## Data Availability

The data used to support the findings of this study are available from the corresponding author upon request.

## Supporting Information

Supporting Information Figure S1: HPLC chromatograms of monosaccharide composition of *Lycium barbarum* polysaccharide (LBP). Figure S2: Schematic representations of the animal experimental procedures. Figure S3: Food and water intake monitoring during the 14-day subacute toxicity experiment. Table S1: Primer sequences used for quantitative real-time PCR. Table S2: Effect of LBP on behaviors and clinical signals observed in the modified Irwin test during the subacute toxicology study in C57BL/6 mice. Table S3: Hematological parameters in the LBP-treated and control groups at the end of the subacute toxicology experiment (14-day). Table S4: Biochemical parameters in the LBP-treated and control group at the end of the subacute toxicology experiment (14-day).
