# Supplementary material for: The Beneficial Effect of Lycium barbarum Polysaccharides on Insulin Resistance and Hepatic Glucose Production in Diabetes
Source: Anal Cell Pathol (Amst). 2025 Sep 15;2025:3176140. doi: 10.1155/ancp/3176140 (PMC12454928; doi:10.1155/ancp/3176140)
Supplement: Supporting Information — Figure S1: HPLC chromatograms of monosaccharide composition of Lycium barbarum polysaccharide (LBP). Figure S2: Schematic representations of the animal experimental procedures. Figure S3: Food and water intake monitoring during the 14-day subacute toxicity experiment. Table S1: Primer sequences used for quantitative real-time PCR. Table S2: Effect of LBP on behaviors and clinical signals observed in the modified Irwin test during the subacute toxicology study in C57BL/6 mice. Table S3: Hematological parameters in the LBP-treated and control groups at the end of the subacute toxicology experiment (14-day). Table S4: Biochemical parameters in the LBP-treated and control group at the end of the subacute toxicology experiment (14-day). [file 3176140.f1.docx]

**The beneficial effects of** *Lycium barbarum* **polysaccharides on insulin resistance and hepatic glucose production in diabetes**

Fengqi Wan^a^, Jiong Dang^a^, Shan Huang^a^, Jiao Cai^a^, Yu Lu^a^, Jiaxin Wu^b^, Jing Wang^a^, Liang Ma^c*^

1. The Second Hospital & Clinical Medical School, Lanzhou University, Lanzhou, China
2. School of Pharmacy, Lanzhou University, Lanzhou, China
3. Institute of Modern Physics, Chinese Academy of Sciences, Lanzhou, China

***Corresponding authors:**

**Liang Ma,** research associate, Institute of Modern Physics, Chinese Academy of Sciences, No.509 Nanchang Road, Lanzhou, China. E-mail: [maliang@impcas.ac.cn](mailto:maliang@impcas.ac.cn)

The fruit of *Lycium barbarum* L. (*LB*L.) was harvested from the coteau of Jingyuan, Gansu Province, China. *Lycium barbarum* polysaccharides (LBP) was obtained from the fruits of *LB*L. using the method of water extraction and ethanol precipitation in our laboratory. The optimal conditions were determined using 3D response surface plots and the contour plots derived from mathematical models. LBP was optimally extracted at a temperature of 93.2℃, an extraction time of 3.9 hours, and a liquid-to-solid ratio of 36.6:1. Under these conditions, the experimental yield of LBP was 4.28%. High-performance liquid chromatography (HPLC) analysis revealed that LBP is composed of arabinose, galactose, glucose, galacturonic acid, mannose, and rhamnose at a molar ratio of 12.25 : 8.66 : 7.66 : 2.86 : 1.70 : 1.00.

**
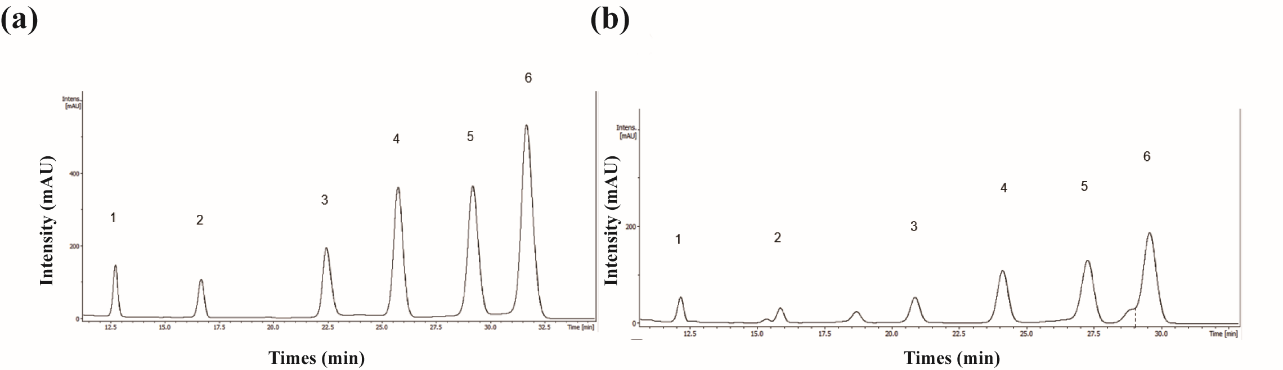
Figure S1. HPLC chromatograms of monosaccharide composition of** *Lycium barbarum* **polysaccharide (LBP)** ^[1,2]^**.** (a) PMP (1-Phenyl-3-methyl-5-pyrazolone) derivatives of six standard monosaccharide, (b) the monosaccharide composition of LBP. 1-Mannose, 2-Rhamnose, 3-Galacturonic acid, 4-Glucose, 5-Galactose, 6-Arabinose.


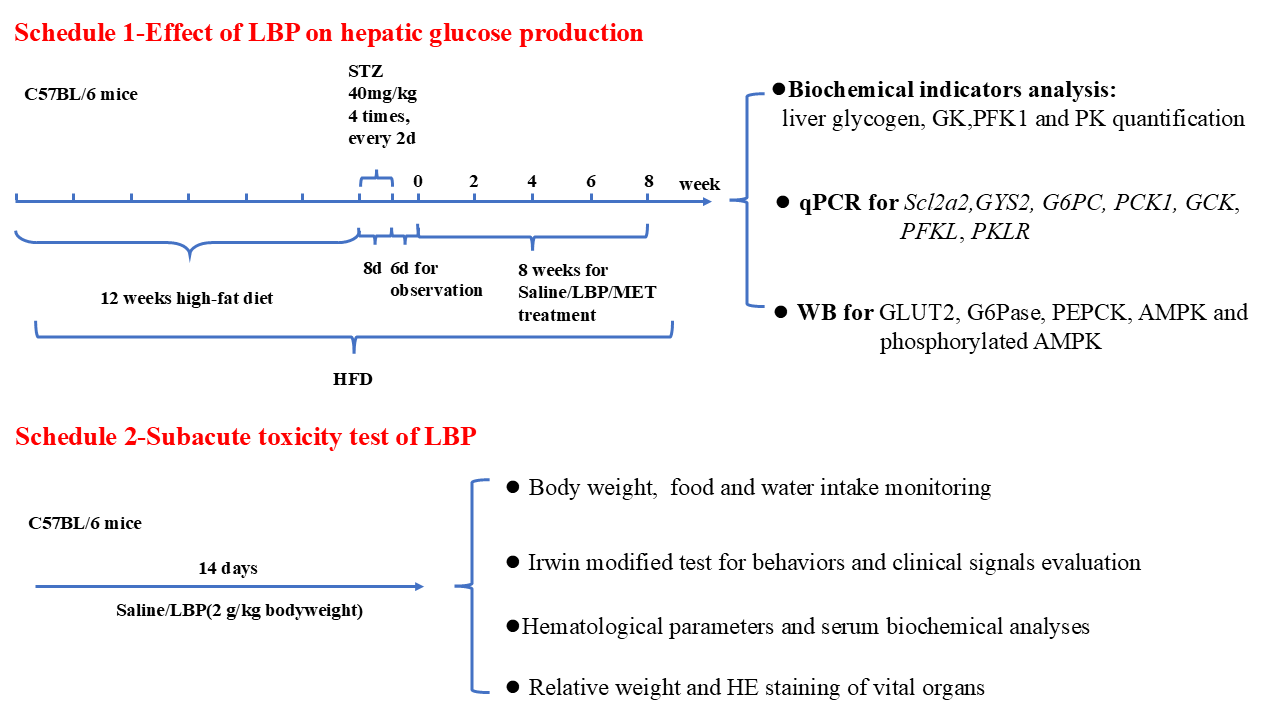


**Figure S2 Schematic representations of the animal experimental procedures.**


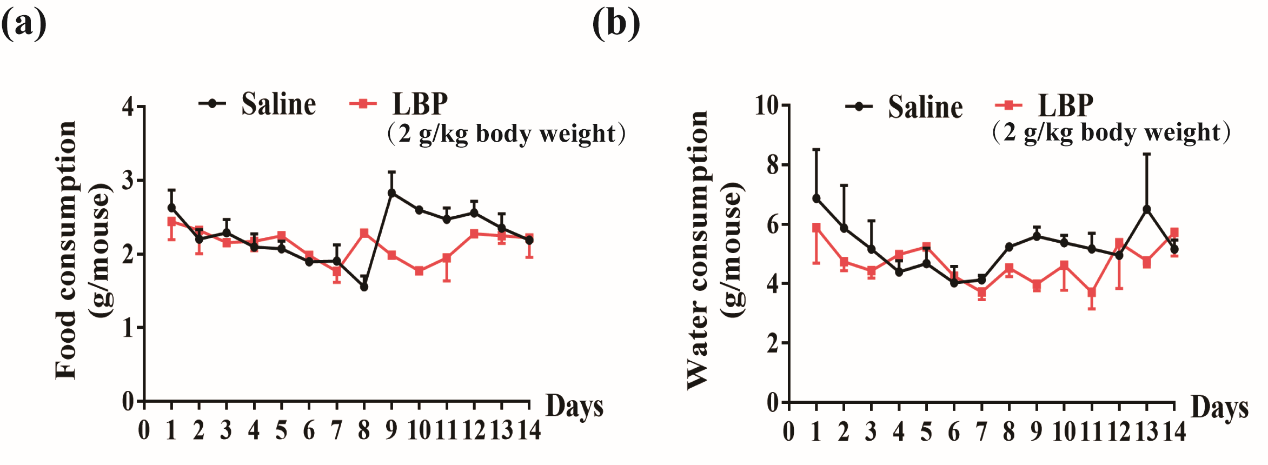


**Figure S3 Food and water intake monitoring during the 14-day subacute toxicity experiment**. (a) Food consumption, (b) Water consumption. Values are means ± SD, 10 mice of each group were divided into two cages and the value is the mean food or water consumption per mouse.

**Table S1 Primer sequences used for quantitative real-time PCR**

| Name | Sequences (5'-3') |
| --- | --- |
| *GYS2-F(mus)* | CAGCTTGACAAGTTCGACATTG |
| *GYS2-R(mus)* | GAGTCACTACATCAGGCTTCCTCT |
| *Slc2a2-F(mus)* | GGCATCAGCCAGCCTGTGTA |
| *Slc2a2-R(mus)* | CATGCCAATCATCCCGGTTAG |
| *G6PC-F(mus)* | ATCAATCTCCTCTGGGTGGC |
| *G6PC-R(mus)* | TGTTGCTGTAGTAGTCGGTGTCC |
| *PCK1-F(mus)* | ACTGTTGGCTGGCTCTCACT |
| *PCK1-R(mus)* | TACATGGTGCGGCCTTTC |
| *PFKL-F(mus)* | CAATGGAGAGTTGTGATCGC |
| *PFKL-R(mus)* | GGCCTTTAAGTCATGGATGTTG |
| *PKLR-F(mus)* | CATCATTGCCACCATCGG |
| *PKLR-R(mus)* | GATGGACTCTGCATGGTACTCAT |
| *GCK-F(mus)* | ACCCCAGAAGGCTCAGAAGT |
| *GCK-R(mus)* | GTCAAAGAGCATCTCCGCAGT |
| *β-Actin-F(mus)* | GTGCTATGTTGCTCTAGACTTCG |
| *β-Actin-R(mus)* | ATGCCACAGGATTCCATACC |

**Table S2 Effect of LBP on behaviors and clinical signals observed in the modified Irwin test during the subacute toxicology study in C57BL/6 mice.**

| Treatment |  | **Control** | | | | | | | | | | | | | | **LBP（2 g/kg bodyweight）** | | | | | | | | | | | | | | |
| --- | --- | --- | --- | --- | --- | --- | --- | --- | --- | --- | --- | --- | --- | --- | --- | --- | --- | --- | --- | --- | --- | --- | --- | --- | --- | --- | --- | --- | --- | --- |
| Observation Time (Days)  天 | | 1 | 2 | 3 | 4 | 5 | 6 | 7 | 8 | 9 | 10 | 11 | 12 | 13 | 14 | 1 | 2 | 3 | 4 | 5 | 6 | 7 | 8 | 9 | 10 | 11 | 12 | 13 | 14 |  |
| Death |  | 0 | 0 | 0 | 0 | 0 | 0 | 0 | 0 | 0 | 0 | 0 | 0 | 0 | 0 | 0 | 0 | 0 | 0 | 0 | 0 | 0 | 0 | 0 | 0 | 0 | 0 | 0 | 0 |  |
| Excitation | Convulsions | 0 | 0 | 0 | 0 | 0 | 0 | 0 | 0 | 0 | 0 | 0 | 0 | 0 | 0 | 0 | 0 | 0 | 0 | 0 | 0 | 0 | 0 | 0 | 0 | 0 | 0 | 0 | 0 |  |
|  | Increased activity | 0 | 0 | 0 | 0 | 0 | 0 | 0 | 0 | 0 | 0 | 0 | 0 | 0 | 0 | 0 | 0 | 0 | 0 | 0 | 0 | 0 | 0 | 0 | 0 | 0 | 0 | 0 | 0 |  |
|  | Increased fear/startle | 0 | 0 | 0 | 0 | 0 | 0 | 0 | 0 | 0 | 0 | 0 | 0 | 0 | 0 | 0 | 0 | 0 | 0 | 0 | 0 | 0 | 0 | 0 | 0 | 0 | 0 | 0 | 0 |  |
|  | Increased abdominal muscle tone | 0 | 0 | 0 | 0 | 0 | 0 | 0 | 0 | 0 | 0 | 0 | 0 | 0 | 0 | 0 | 0 | 0 | 0 | 0 | 0 | 0 | 0 | 0 | 0 | 0 | 0 | 0 | 0 |  |
| Motor | Scratching | 0 | 0 | 0 | 0 | 0 | 0 | 0 | 0 | 0 | 0 | 0 | 0 | 0 | 0 | 0 | 0 | 0 | 0 | 0 | 0 | 0 | 0 | 0 | 0 | 0 | 0 | 0 | 0 |  |
|  | Motor incoordination | 0 | 0 | 0 | 0 | 0 | 0 | 0 | 0 | 0 | 0 | 0 | 0 | 0 | 0 | 0 | 0 | 0 | 0 | 0 | 0 | 0 | 0 | 0 | 0 | 0 | 0 | 0 | 0 |  |
|  | Loss of traction | 0 | 0 | 0 | 0 | 0 | 0 | 0 | 0 | 0 | 0 | 0 | 0 | 0 | 0 | 0 | 0 | 0 | 0 | 0 | 0 | 0 | 0 | 0 | 0 | 0 | 0 | 0 | 0 |  |
| Sedation | Decreased activity | 0 | 0 | 0 | 0 | 1 | 0 | 0 | 0 | 0 | 0 | 0 | 0 | 0 | 0 | 0 | 0 | 0 | 0 | 0 | 0 | 0 | 0 | 0 | 0 | 0 | 0 | 0 | 0 |  |
|  | Decreased reactivity to touch | 0 | 0 | 0 | 0 | 0 | 0 | 0 | 0 | 0 | 0 | 0 | 0 | 0 | 0 | 0 | 0 | 0 | 0 | 0 | 0 | 0 | 0 | 0 | 0 | 0 | 0 | 0 | 0 |  |
|  | Decreased abdominal muscle tone | 0 | 0 | 0 | 0 | 0 | 0 | 0 | 0 | 0 | 0 | 0 | 0 | 0 | 0 | 0 | 0 | 0 | 0 | 0 | 0 | 0 | 0 | 0 | 0 | 0 | 0 | 0 | 0 |  |
| Pain | Writhing | 0 | 0 | 0 | 0 | 0 | 0 | 0 | 0 | 0 | 0 | 0 | 0 | 0 | 0 | 0 | 0 | 0 | 0 | 0 | 0 | 0 | 0 | 0 | 0 | 0 | 0 | 0 | 0 |  |
|  | Analgesia | 0 | 0 | 0 | 0 | 0 | 0 | 0 | 0 | 0 | 0 | 0 | 0 | 0 | 0 | 0 | 0 | 0 | 0 | 0 | 0 | 0 | 0 | 0 | 0 | 0 | 0 | 0 | 0 |  |
| Autonomic | Exophthalmia | 0 | 0 | 0 | 0 | 0 | 0 | 0 | 0 | 0 | 0 | 0 | 0 | 0 | 0 | 0 | 0 | 0 | 0 | 0 | 0 | 0 | 0 | 0 | 0 | 0 | 0 | 0 | 0 |  |
|  | Myosis/Mydriasis | 0 | 0 | 0 | 0 | 0 | 0 | 0 | 0 | 0 | 0 | 0 | 0 | 0 | 0 | 0 | 0 | 0 | 0 | 0 | 0 | 0 | 0 | 0 | 0 | 0 | 0 | 0 | 0 |  |
|  | Defecation/diarrhea | 0 | 0 | 0 | 0 | 0 | 0 | 0 | 0 | 0 | 0 | 0 | 0 | 0 | 0 | 0 | 0 | 0 | 0 | 0 | 0 | 0 | 0 | 0 | 0 | 0 | 0 | 0 | 0 |  |
|  | Salivation /Lacrimation | 0 | 0 | 0 | 0 | 0 | 0 | 0 | 0 | 0 | 0 | 0 | 0 | 0 | 0 | 0 | 0 | 0 | 0 | 0 | 0 | 0 | 0 | 0 | 0 | 0 | 0 | 0 | 0 |  |
| Respiration | Increased | 0 | 0 | 0 | 0 | 0 | 0 | 0 | 0 | 0 | 0 | 0 | 0 | 0 | 0 | 0 | 0 | 0 | 0 | 0 | 0 | 0 | 0 | 0 | 0 | 0 | 0 | 0 | 0 |  |
|  | Decreased | 0 | 0 | 0 | 0 | 0 | 0 | 0 | 0 | 0 | 0 | 0 | 0 | 0 | 0 | 0 | 0 | 0 | 0 | 0 | 0 | 0 | 0 | 0 | 0 | 0 | 0 | 0 | 0 |  |

Note: “0” means there was no mouse has such behavior and clinical signal, “1” means one mouse has such characteristic.

**Table S3. Hematological parameters in the LBP-treated and control groups at the end of the subacute toxicology experiment (14-day)**

|  | **Control** | **LBP**  **（2 g/kg bodyweight）** | *p* |
| --- | --- | --- | --- |
| WBC（10^9^/L） | 2.53 ± 0.77 | 2.36 ± 0.69 | 0.6062 |
| Neutrophil（10^9^/L） | 0.67 ± 0.60 | 0.48 ± 0.47 | 0.4148 |
| Lymphocytes（10^9^/L） | 1.21 ± 0.77 | 1.62 ± 0.71 | 0.2144 |
| Monocytes（10^9^/L） | 0.09 ± 0.06 | 0.04 ± 0.04 | 0.0980 |
| Eosinophil（10^9^/L） | 0.11 ± 0.09 | 0.09 ± 0.06 | 0.5651 |
| Neutrophil Ratio | 0.25 ± 0.14 | 0.21 ± 0.13 | 0.3219 |
| Monocytes Ratio | 0.04 ± 0.02 | 0.03 ± 0.03 | 0.2347 |
| Eosinophil Ratio | 0.05 ± 0.03 | 0.04 ± 0.04 | 0.5645 |
| RBC（10^12^/L） | 9.22 ± 0.62 | 9.55 ± 0.60 | 0.2187 |
| Hematocrit（L/L） | 0.47 ± 0.03 | 0.50 ± 0.03 | 0.0751 |
| Hemoglobin（g/L） | 138.70 ± 8.90 | 141.20 ± 8.45 | 0.5913 |
| Platelets（10^9^/L） | 800.90 ± 126.81 | 789.18 ± 156.16 | 0.8532 |

Note：WBC (white blood cells), RBC (red blood cells). Values are means ± SD (n = 10 mice per group). The *p* value were comparisons made between Control (Saline) and LBP (2 g/kg body weight) groups.

**Table S4. Biochemical parameters in the LBP-treated and control groups at the end of the subacute toxicology experiment (14-day)**

|  | **Control** | **LBP**  (2 g/kg body weight ) | *p* |
| --- | --- | --- | --- |
| Gluocse (mM) | 9.55 ± 1.21 | 8.96 ± 0.72 | 0.5089 |
| Total cholesterol (mM) | 1.85 ± 0.11 | 1.82 ± 0.19 | 0.8396 |
| Triglyceride (mM) | 0.86 ± 0.10 | 0.58 ± 0.01 | 0.0003^***^ |
| Total protein (g/L) | 50.87 ± 2.98 | 49.43 ± 3.37 | 0.6107 |
| Albumin (g/L) | 29.33 ± 0.85 | 29.13 ± 1.25 | 0.8300 |
| Globulin (g/L) | 21.53 ± 2.17 | 20.30 ± 2.13 | 0.5212 |
| Urea (mM) | 7.63 ± 1.07 | 6.66 ± 1.49 | 0.4112 |
| Creatinine (μM) | 12.98 ± 0.24 | 13.80 ± 1.30 | 0.2595 |
| ALT (U/L) | 33.28 ± 5.04 | 25.45 ± 4.17 | 0.0150^*^ |
| AST (U/L) | 89.00 ± 18.36 | 66.50 ± 12.32 | 0.0319^*^ |
| ALT/AST | 0.39 ± 0.10 | 0.39 ± 0.10 | 0.8182 |
| Total bilirubin (μM) | 2.43 ± 0.48 | 2.67 ± 0.62 | 0.4823 |
| K (mM) | 4.66 ± 0.35 | 4.94 ± 0.79 | 0.6011 |
| Na (mM) | 141.50 ± 3.49 | 147.50 ± 3.95 | 0.1199 |
| Ca (mM) | 2.20 ± 0.13 | 2.19 ± 0.13 | 0.9150 |
| Mg (mM) | 1.41 ± 0.03 | 1.40 ± 0.19 | 0.9306 |

Note: Values are means ± SD (n =5-7 mice per group). The comparisons were made as ‘*’- Control (Saline) *vs* LBP (2 g/kg body weight), ^*^*p* ＜ 0.05, ^***^*p* ＜ 0.001.

1. Wu J, Ma L, Chen T, et al. Optimization of extraction technology and chromatographic analysis of monosaccharides composition of Lycium barbarum polysaccharides*.* *Western J Traditional Chinese Medicine* (Chinese Journal), 2021, 34: 44-49.
2. Wan F, Ma F, Wu J, et al. Effect of *Lycium barbarum* polysaccharide on decreasing serum amyloid A3 expression through inhibiting NF-κB activation in a mouse model of diabetic nephropathy*.* *Anal Cell Pathol*, 2022, 2022: 7847135.
